# Key Worker–Mediated Enhancement of Physical Health in First Episode Psychosis: Protocol For a Feasibility Study in Primary Care

**DOI:** 10.2196/13115

**Published:** 2019-07-10

**Authors:** Geoff McCombe, Aine Harrold, Katherine Brown, Liam Hennessy, Mary Clarke, David Hanlon, Sinead O'Brien, John Lyne, Ciaran Corcoran, Patrick McGorry, Walter Cullen

**Affiliations:** 1 School of Medicine University College Dublin Dublin Ireland; 2 Dr Steeven's Hospital Health Service Executive Dublin Ireland; 3 Health Service Executive Dublin Ireland; 4 Health Service Executive Cork Ireland; 5 Royal College of Surgeons Ireland Dublin Ireland; 6 North Dublin Mental Health Services Dublin Ireland; 7 Health Service Executive Westmeath Ireland; 8 University of Melbourne Melbourne Australia; 9 The National Centre of Excellence in Youth Mental Health Orygen Melbourne Australia; 10 Health Sciences Centre Belfield Ireland

**Keywords:** psychosis, review, health, primary care, secondary care

## Abstract

**Background:**

Studies have demonstrated that, for patients with psychosis, a majority of the decline in health status and functioning emerges during the first few years after the onset of illness. This knowledge led to the development of specialized early intervention services (EISs) targeting patients experiencing their first episode of psychosis. The central component of EISs is often assertive case management delivered by a multidisciplinary team, where an appointed key worker is responsible for coordinating treatment and delivering various psychosocial interventions to service users.

**Objective:**

This paper outlines the protocol for a feasibility study examining how key workers may enhance physical health by supporting integration between primary and secondary care.

**Methods:**

Semistructured interviews were conducted with key stakeholder groups (General Practitioners and health care professionals working in mental health services). The interviews informed the development of the complex intervention involving a longitudinal pre-post intervention in 8 general practices in 2 regions in Ireland (one urban and one rural). Patients with first episode psychosis (FEP) will be identified from clinical records at general practices and mental health services.

**Results:**

Baseline and follow-up data (at 6 months) will be collected, examining measures of feasibility, acceptability, and intervention effect size.

**Conclusions:**

Study findings will inform future practice by examining feasibility of key workers enhancing physical health through improved interaction between primary and secondary care. By identifying issues involved in enhancing recruitment and retention, as well as the likely effect size, the study will inform a future definitive intervention.

**International Registered Report Identifier (IRRID):**

DERR1-10.2196/13115

## Introduction

### Background

Psychosis is a clinical syndrome that affects several domains, including affective, cognitive, motivational, sensory, and social functioning. Psychosis can manifest in a variety of symptoms including positive symptoms (eg, delusions and hallucinations), negative symptoms (eg, reductions in motivation, volition, and emotion experience or expression), declines in cognitive and social functioning, and disorganized speech and behavior [1]. Psychosis often manifests as a symptom of psychotic-spectrum disorders, which include schizophrenia-spectrum disorders (eg, schizophrenia and schizoaffective disorder) and affective disorders with psychotic features (eg, bipolar disorder with psychotic features) [2]. Psychotic-spectrum disorders are associated with severe difficulties in psychiatric, physical, and functional well-being. Each year it is estimated that 1500 people develop a first episode of psychosis in Ireland. The implications of this issue for population health are considerable. Psychosis usually develops in late adolescence or early adulthood, a critical phase of our life cycle in terms of personal, academic, and economic development. In addition, the personal, familial, and societal costs of psychosis are considerable [3-7].

It has been widely documented that individuals living with psychotic-spectrum disorders have high mortality rates [8,9], and patients with severe mental health disorders, such as psychosis, have a life expectancy that is 10 to 25 years lower than age-matched peers in the general population [8,10-16]. This is partly due to higher rates of suicide, which has been found to be up to 12 times greater in this group than the general population [17,18]. However, suicide accounts for only a fraction of this reduced life expectancy, and the majority is due to the higher rates of cardiovascular, pulmonary, and infectious diseases found in this population [17,19]. There is now clear evidence that weight gain, cardiovascular risk, and metabolic disturbance commonly appear early in the course of emerging psychosis and are potentially modifiable [20]. Psychosis is associated with unhealthy lifestyle choices such as high rates of alcohol, drug and tobacco use, poor nutrition, and low activity levels [21-28]. Heavy smoking is 2 to 6 times more common among people with schizophrenia. Obesity exists in 45% to 55% of people with schizophrenia, diabetes in 10% to 15%, and hypertension in 19% to 58% [29-31]. Individuals with severe mental illness receive poorer medical care for their physical health problems than do members of the general population [11,32]. These factors result in poorer health outcomes and mortality in people with psychosis [33,34].

In the Early Intervention in Psychosis (EIP) model of care, First Episode Psychosis (FEP) is defined as “psychotic symptoms that have lasted at least a week (ie, hallucinations and/or delusions with/without evidence of thought disorder for at least seven consecutive days) leading to distress or disruption to functioning” [35] and that continues throughout the entire critical period [36]. The critical period is defined as the first 5 years for a subset of people [35]. For all others, there is international consensus that treatment should continue for at least 2 years [37].

The first few years of psychotic-spectrum disorders are likely to be a *critical period* in which the provision of targeted, phase-specific intervention could dramatically improve the usual course of psychotic-spectrum disorders [38]. Research has demonstrated that a majority of the decline in health status occurs in the first few years following the onset of psychosis [39]. Furthermore, individuals earlier in the course of a psychotic disorder may be more responsive to both pharmacological and psychosocial treatments than those with a more long-standing illness.

In recent years, an international consensus has identified that most people who develop psychosis are unwell for a considerable period of time before seeking help [4,32,40,41]. This time period is called the *duration of untreated psychosis* and it is crucial because the longer individuals with psychosis remain undiagnosed and untreated, the greater the opportunity for adverse physical, psychological, and social outcomes. Reducing the duration of untreated psychosis and ensuring people receive treatment that is specific to the early phase of the illness are associated with improved physical and social outcomes. Therefore, early detection and optimal early treatment in people experiencing their first episode of psychosis have been emphasized as a best practice in FEP literature in recent years [42]. Pioneering work in Melbourne that established *Early Intervention* teams to work with individuals during a first episode of psychosis demonstrated considerable benefits in terms of health gain and satisfaction to the family and economically to the state [43-45] and this model of care has been replicated worldwide [46-48]. These services are characterized by holistic, multimodal, and phase-specific treatment of patients with FEP, typically centred around assertive case management with access to a comprehensive range of pharmacological and psychosocial interventions [49].

Primary care has a key role in the care of patients who experience FEP, and effective links between secondary and primary care have been a key feature of Ireland’s FEP Early Intervention Services (EISs) [50,51]. EISs seek to enhance the outcome trajectories of psychotic-spectrum disorders [38,52,53] by focusing on early detection of new cases [54,55], shortening delays to effective treatment [54,56-58] and providing comprehensive and timely treatment to patients with FEP throughout the entire critical period [58,59]. Early intervention programs generally engage in some form of assertive community treatment [60-62], which attempts to treat patients in the community instead of making use of inpatient services [63]. Therefore, the presence of a primary care point of contact between service users and mental health services is an important factor for many EISs. Key workers have been identified as a key strategy to support patient engagement with mental health services and this is especially the case for patients with psychosis [64,65]. Furthermore, Ireland’s Health Services Executive, specifically the Clinical Programme (Early Intervention for First Episode Psychosis [EIP]) has identified EIP key workers as a key mechanism to enhance links between primary and secondary care and to improve physical health (Early Intervention Services for Psychosis, Submission to the National Clinical Programme HSE by Early Intervention Working Group of the College of Psychiatrists of Ireland, 2015).

Key workers can be from a range of mental health clinical backgrounds but need to be of a certain level of seniority. They must also be adequately trained and maintain competencies in early intervention, skills including assessment of psychosis, relapse prevention, and family psychoeducation and assessment of suicide or violence risk [66]. Key workers can serve as the consistent point of contact between the service user (and family or carers), the EISs, and other agencies involved, provide basic psychosocial interventions, and ensure the organization of individual care plans and service transfers for their patients. EIP key workers have an important role in enhancing initial diagnosis and subsequent treatment for FEP. Increased liaison between primary and secondary care improves the clinical effectiveness and cost-effectiveness of detection of people with or at high risk of developing FEP [67]. Although EISs provide formal structured professional support for service users, the role of EIP key workers from *assessment and engagement* through to the *long-term successful delivery of effective treatments* is also crucial [68]. There is increasing consensus that EIP key workers for those suffering from psychosis are seen as important health care *resources* [69]. With a general consensus that integrated approaches to health care are likely to enhance outcomes, and in the case of patients experiencing FEP, a recognition of the potential of EIP key workers to promote this goal, this is an ideal context in which to examine how EIP key workers can enhance the capacity of primary and secondary care to collaborate to enhance physical health outcomes for people who experience an FEP. This question has not been addressed internationally and such knowledge is needed to inform service development, and especially integrated care, thus reducing the global burden of chronic disease among people with severe enduring mental illness.

### Objective

This study aims to inform health policy in Ireland and internationally by conducting a mixed-methods study examining how key workers might enhance integration between primary and secondary care to improve outcomes for patients with FEP. Outcomes of interest include general and mental health outcomes, substance use disorders, and chronic illness and multimorbidity prevalence. We will examine the feasibility, acceptability, and likely efficacy of a key worker–led intervention in a *real-world* clinical setting, thereby informing future definitive interventions in the area.

## Methods

This project design was informed by the *MRC Framework for the Design and Evaluation of Complex Interventions to Improve Health* [70], which suggests the phased development of health interventions. The study design involved a mixed methodology in primary and secondary care in Ireland, with 2 sequential phases.

### Study Design and Setting

A longitudinal pre-post intervention in 8 general practices in 2 regions in Ireland (one urban and one rural), in which patients with FEP, will be identified from clinical records in general practice (using a previously developed software tool [71]) and in mental health services. Baseline and follow-up data (at 6 months) will be collected on a number of measures of patients’ physical and mental health.

### Intervention Development and Design

**Figure 1:**
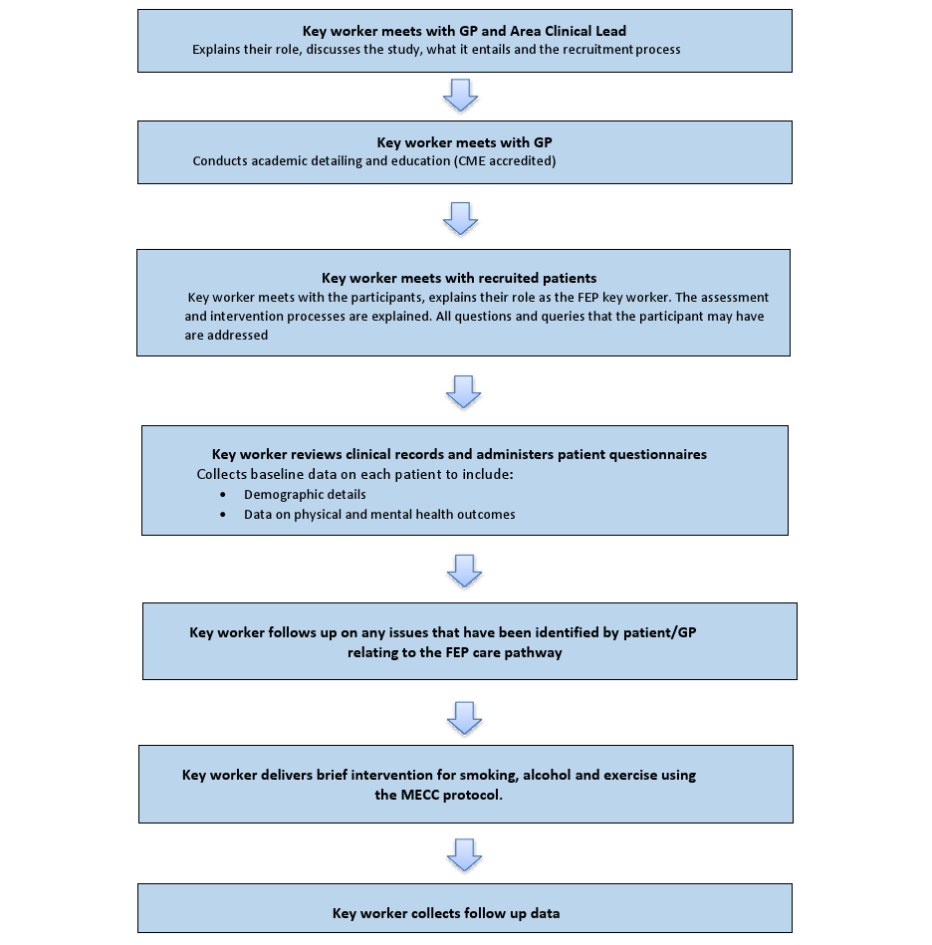
Key worker intervention.

Semistructured interviews will be conducted with GPs and health care professionals, such as psychiatrists and nurses, working in mental health services (n=16) to inform the *complex intervention* which will consist of the following:

- Academic detailing.
- Education and training of GPs.
- Key worker:
  - To optimize (bidirectional) communication between primary and secondary care with regard to physical health issues requiring follow-up.
  - To deliver brief interventions for problem alcohol use/tobacco smoking.
  - To identify community-based health agencies (eg, primary care team members, nongovernmental organizations, and third sector) who can assist with preventative health interventions (see Figure 1).

### Approach to Sampling and Recruitment

The clinical lead in each of Ireland’s mental health service catchment areas will be invited to participate, and expressions of interest will be sought to participate in this feasibility study. From these expressions of interest, one urban and one rural service will be identified.

At both sites, all general practices will be eligible to participate in the study. From those who express an interest in participating, 4 practices will be selected using stratified sampling, to be representative in terms of practice size and location. Sampled GPs will be contacted about their participation, given further information on the study (eg, what their involvement will entail), and consulted about patient recruitment. The research team will telephone those not replying. Each practice will be visited by the principal investigator/lead researcher and provided with information about the research program.

**Figure 2:**
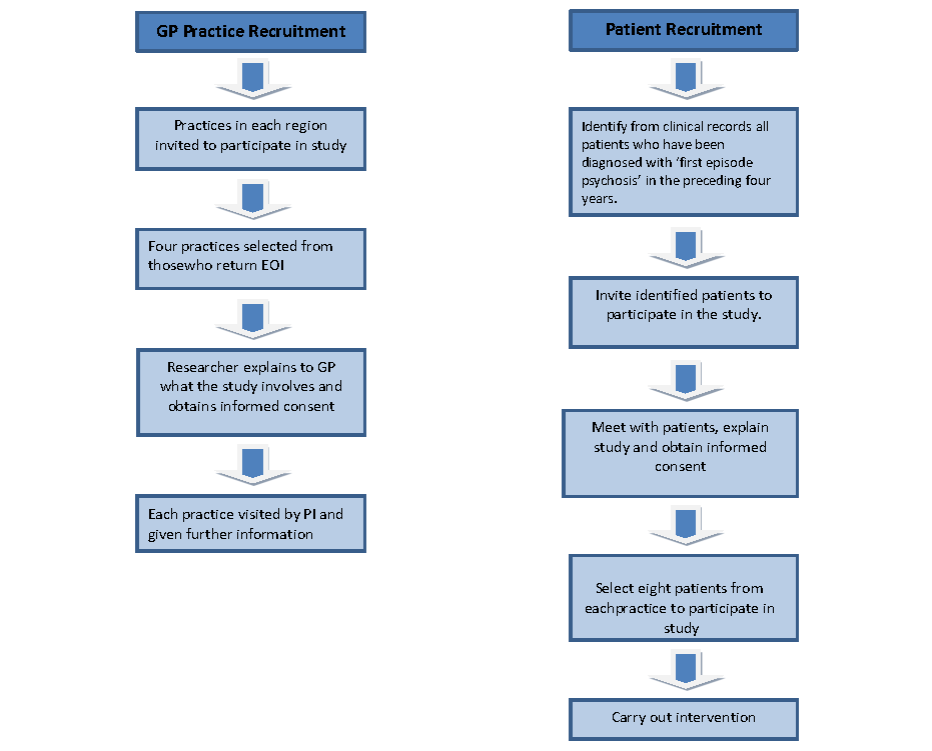
CONSORT diagram—practice and patient recruitment.

At each participating practice, all patients who have been diagnosed with *FEP* in the preceding 4 years will be identified from clinical records (at the general practice or at the mental health service) and invited to participate in the study. Potential participants will be given written information on the study. Those interested in participating will be invited to meet a researcher who will be at the practice during the recruitment period. At this meeting, interested patients will be given further information on the study and will have an opportunity to ask the researcher questions. If they consent to participate, patients will be asked to sign a consent form. In total, 8 participants will be selected to participate from each practice (see Figure 2).

### Sample Size

Semistructured interviews will be conducted with 16 health care professionals working in mental health services (n=16) to inform the *complex intervention*. Although it is difficult to predict the number of participants required to reach data saturation, our previous qualitative work [72,73] has indicated that 12 to 16 verbatim are required.

The goal of this feasibility study is to estimate rates of recruitment, consent, retention and response, methodological procedures, and issues. In addition, estimation of the parameters of likely primary outcome measures would allow the sample size of a definitive trial to be determined. With an average of 2 patients presenting to a GP with an FEP each year [74], that is, 8 per 4 years, and 8 practices in total across the 2 sites, we consider the sample size will be sufficient to estimate the actual recruitment and retention rates for a sample of patients recruited in primary care and provide data on acceptability of study processes and outcome measures which will inform a future definitive trial.

### Data Collection

At baseline, demographic details and data on physical and mental health outcomes will be collected by reviewing clinical records and by participants completing study instruments at recruitment (baseline) and 3 months post intervention. Baseline and follow-up data will be collected on the following:

- Mental disorders, using Primary Care Evaluation of Mental Disorders/Patient Health Questionnaire [75].
- Substance use disorders (Alcohol, Smoking, Substance Involvement Screening Test) [76].
- General health status (SF-12) [77].
- Chronic illness and general medical morbidity (ie, clinical records review using a structured instrument previously developed by our group for morbidity surveys among problem drug users attending general practice [78].)
- Cardiometabolic risk, using body composition, blood pressure, and blood samples.

### Qualitative Evaluation

To explore study participants’ experience of the intervention, 6 to 8 health care professionals in participating practices and 6 to 8 patients will be interviewed in depth on the question *your experience of and satisfaction with the complex intervention, how can primary/secondary care work collaboratively to enhance physical health for patients with FEP*. Interviews will be conducted with a semistructured questionnaire (with open questions), in person or by telephone, as preferred by the participant. The conversation will be recorded digitally, and answers to the structured questions will be recorded. Each interview will be transcribed verbatim, following which the transcript will be reviewed by the researchers for accuracy.

### Data Analysis

At baseline and follow-up, descriptive statistics will be estimated with regard to key feasibility variables, that is, as follows:

- Practice recruitment rate—percentage of invited practices who express an interest in participating.
- Patient recruitment rate—percentage of invited patients who participate.
- Prevalence of cardiovascular disease, diabetes, and tobacco and substance use.
- Practice/patient retention rates.

The IBM SPSS version 20 statistical package will be used for statistical analysis.

### Qualitative Data Analysis

Thematic analysis will be used to analyze qualitative data. This approach has many benefits for studies such as this which are interpretive in nature, as it is a “method for identifying, analyzing and reporting patterns (themes) within data” [79]. The process of thematic analysis is concerned with the basic to advanced encoding of data. The codes are subsequently developed to themes. This flexible approach can also be seen in how themes identified at one level can help the researcher describe their observations and at a more advanced level allow the researcher to interpret aspects of the phenomenon under study [79]. The qualitative research software NVivo version 8 will be used to facilitate the coding of these data. The analysis will follow a *5-Step Analysis* approach whereby data are reviewed, examined, coded, and themes generated and defined [79]. To achieve validity in the coding/analysis of data, 2 reviewers will code data independently and inter-rater reliability measures will be computed based on this coding. Coding consistency will be maintained throughout the coding process and will be reviewed by regular meetings between researchers and the principal investigator. The findings will be compared with other study findings (validity and credibility). The researchers will present the findings to participants to determine if the study findings reflect their experience of the topic under study (member checking). Illustrative quotes will be used to emphasize points made by the participants **.**

### Ethical Considerations

Ethical considerations and safeguards include the following:

- Informed consent and consenting capacity: all potential participants (GPs and patients) will be given written information on the study and the model of care being proposed and will be asked to provide written consent that they are happy to participate and that nonparticipation will not compromise their usual care. Participation in the study will be on a voluntary basis. No inducements to participate will be offered.
- Confidentiality: Any data/personal details that could potentially reveal the identity of individuals will be removed. Only anonymized, deidentified information will leave the practice of origin. To allow follow-up, an alphanumeric code will be assigned to each participant’s data; a database will be maintained on a password-protected database. The list will be kept separately from patient data but will indicate the medical record number of each participant and the alphanumeric code. All research data will be stored on a password-protected desktop computer at the host organization. Study participants will be invited to give permission to have their name, address, and contact details held by the research team to facilitate their receiving a synopsis of the study findings on publication and to be contacted for follow-up data collection. All data will be stored securely at the host institution.
- Clinical governance does no harm: it is possible that participating in the study may raise health-related issues for participants and may identify a health issue that requires clinical intervention. Therefore, all participants will be advised to speak with their doctor if participating in the study has raised any such issues. Furthermore, only patients who health care professionals deem able to participate will be asked to take part.
- General Data Protection Regulation (GDPR): GDPR compliance will be adhered to in terms of the following:
  - Data privacy rights—participants will have the right to request information about their data throughout the research process.
  - Transfer of data—participants will be informed about the circumstances under which their data may be transferred and safety measures which will be taken to protect the data (eg, data are encoded).
  - Retention of data—patients will be informed how long their data will be stored.

Application will be made to the Health Service Executive, Irish College of General Practitioners (GPs) and University College Dublin Research Ethics Committees.

## Results

The study findings have the potential to provide important information on how key workers might enhance collaboration between primary and secondary care to improve outcomes for patients with FEP.

## Discussion

### Strengths and Limitations

This study is the first study to examine how key workers might enhance collaboration between primary and secondary care to improve outcomes for patients with FEP. It will provide important information to enhance scientific understanding of the role of key workers in improving health outcomes for patients with FEP. It will provide key information to inform health policy and service development in Ireland and internationally. However, it may be difficult to extrapolate these results among a high-risk population because of the specificity of the symptomatology in the early phases. This study has the potential to make an important impact on patient care and will provide high-quality evidence to help inform health care professionals on the importance of key workers for FEP patients. The intervention is scalable and, therefore, if found to be feasible and acceptable, it can be readily implemented elsewhere and used to guide policy and service development internationally.

Possible limitations of the study include potential issues of bias and lack of generalizability that may arise from the recruitment process, owing to the likelihood that health care professionals who are more interested in research and innovation will choose to participate. As qualitative data analysis is open to interpretation, there are also potential issues of bias that may arise from data analysis. The use of multiple researchers during the qualitative analysis phase will attempt to reduce this possibility. Despite these potential limitations, this study will provide important information regarding the role of key workers in improving collaboration between primary and secondary care to improve health outcomes for patients with FEP.

### Conclusions

At the end of this study, the feasibility of a clinical intervention, informed by international best practices and local barriers, will be evaluated among a high-risk population. This feasibility study will inform clinical practice by providing initial indications as to how key workers might enhance collaboration between primary and secondary care to improve outcomes for patients with FEP. It will also inform future research on the topic by providing key parameters for the design of a future randomized controlled trial.

## Abbreviations

EIP: Early Intervention in Psychosis
EISs: early intervention services
FEP: First Episode Psychosis
GDPR: General Data Protection Regulation
GP: General Practitioner
